# Cancer immunotherapy: broadening the scope of targetable tumours

**DOI:** 10.1098/rsob.180037

**Published:** 2018-06-06

**Authors:** Jitske van den Bulk, Els ME Verdegaal, Noel FCC de Miranda

**Affiliations:** 1 Department of Pathology, LUMC, Leiden, The Netherlands; 2 Department of Clinical Oncology, LUMC, Leiden, The Netherlands

**Keywords:** neo-antigens, checkpoint blockade, immunotherapy, mutation burden, immunogenicity, combination therapies

## Abstract

Cancer immunotherapy has experienced remarkable advances in recent years. Striking clinical responses have been achieved for several types of solid cancers (e.g. melanoma, non-small cell lung cancer, bladder cancer and mismatch repair-deficient cancers) after treatment of patients with T-cell checkpoint blockade therapies. These have been shown to be particularly effective in the treatment of cancers with high mutation burden, which places tumour-mutated antigens (neo-antigens) centre stage as targets of tumour immunity and cancer immunotherapy. With current technologies, neo-antigens can be identified in a short period of time, which may support the development of complementary, personalized approaches that increase the number of tumours amenable to immunotherapeutic intervention. In addition to reviewing the state of the art in cancer immunotherapy, we discuss potential avenues that can bring the immunotherapy revolution to a broader patient group including cancers with low mutation burden.

## 1. Introduction

The field of cancer immunotherapy has experienced alternating periods of success and failure in the development of cancer therapies. In the late nineteenth century, William Coley treated cancer patients by local injection with bacterial toxins, which provoked anti-tumour immune responses in some patients [1]. In the 1960s, Thomas and Burnet postulated the cancer immune surveillance theory, where the immune system would specifically eliminate malignant cells, most probably through recognition of tumour-associated antigens [2,3]. This was followed by the elucidation of the role of T cells in anti-tumour immune responses which led to the clinical use of the T-cell growth factor interleukin-2 (IL-2). In 1991, IL-2 was approved by the FDA for the treatment of metastatic renal cell carcinoma and, in 1998, for metastatic melanoma. However, IL-2 treatments produced high toxicity and yielded a relatively low response rate, underlining the need to develop improved immunotherapeutic strategies [4,5].

The transition to targeted immunotherapy was made with the development of the hybridoma technology, in 1975, which supported the production of monoclonal antibodies [6]. Rapidly, monoclonal antibody-based treatments were set up and the first FDA approval was obtained for rituximab in 1997 for the treatment of B-cell lymphomas. Rituximab is a genetically engineered monoclonal antibody directed against the CD20 antigen which is ubiquitously expressed in B cells and triggers cell death by antibody-dependent cell-mediated cytotoxicity, complement activation and direct induction of apoptosis [7,8]. In the same decade, chimaeric antigen receptor (CAR) T cells were developed to combine the antigen-binding properties of antibodies with the cytolytic and self-renewal capacity of T cells [9,10]. CAR T cells are genetically engineered to express an extracellular antigen-recognition domain, such as antibody-derived, single-chain variable fragments, coupled to T-cell activation endodomains. The most significant clinical results have been achieved with CD19-targeting CAR T cells in haematological malignancies [11,12].

More recently, a number of antibodies targeting cellular immune checkpoints (e.g. PD-1/PD-L1 and CTLA-4) have been developed to promote the activation of T cells and subsequent tumour control. This treatment strategy has been shown to be particularly effective in tumours with high mutation burden, putting tumour-mutated antigens (neo-antigens) centre stage in cancer immunotherapy [13–19].

## 2. Antigen presentation and cancer immunotherapy

Antigen processing and presentation enables the immune system to monitor cellular processes and to act accordingly upon expression of aberrant/foreign proteins. Human leukocyte antigen (HLA) class I molecules present antigens at the surface of most cells throughout the organism. Such antigens can, theoretically, be derived from most cellular proteins as these are processed by the (immuno) proteasome and broken down to peptides [20]. Subsequently, transporter associated with antigen processing (TAP) proteins mediate the intake of these peptides to the endoplasmic reticulum, where they are loaded onto HLA class I molecules with the aid of several chaperones [21,22]. HLA class I/peptide complexes translocate via the Golgi apparatus to the cell surface where they are exposed to CD8^+^ T cells [23]. Nevertheless, an effective anti-tumour immune response is thought to be initiated by the taking up of tumour antigens by antigen-presenting cells (APCs) which in turn present them, and provide co-stimulatory signals, to both CD4^+^ and CD8^+^ T cells [24]. In order to do so, APCs, particularly dendritic cells, process antigens through an exogenous antigen processing pathway where (tumour) cellular material is phagocytosed and converted into HLA class I- and class II-binding peptides that are presented to CD8^+^ (cross-presentation) and CD4^+^ T cells, respectively [25]. HLA class II expression is also known to occur in some tumour types although its functional significance and how it can be exploited from an immunotherapeutic point of view require further investigation [26,27].

Antigens that are considered to evoke anti-tumour immune responses and which are therefore suitable as immunotherapeutic targets can be divided into three groups: tumour-mutated antigens (or neo-antigens), tumour-associated antigens and cancer-testis antigens [28]. Viral antigens constitute another class of targetable antigens in the context of viral oncogenesis but will not be discussed here. Tumour-associated and cancer-testis antigens are both self-antigens that are differentially expressed in tumour tissues and rarely expressed (or to lower extent) in normal tissues. The stimulation of endogenous T-cell responses against self-antigens can be challenging as auto-reactive T cells are subjected to negative selection in the thymus [29]. Nevertheless, it has been shown that central tolerance can be broken and that immune responses can be generated against self-antigens, analogous to what is observed in autoimmunity [30]. Positive clinical indications have been described for several tumour-associated antigens (e.g. gp100, MART-1) and cancer-testis antigens (e.g. MAGE-A3 and NY-ESO-1) [31–34]. However, subsequent clinical trials were not always able to confirm patient survival benefits and side-effects were regularly observed due to expression of the targeted antigens in healthy tissues [35–37].

Neo-antigens are by definition tumour-specific as they arise from somatic mutations that are not present in healthy tissue. Theoretically, they constitute ideal targets for immunotherapy because no off-target reactivity and central tolerance of T cells are expected [38]. The accumulation of somatic mutations is a hallmark of tumour progression, but only a minority of mutations is under positive selection and, therefore, recurrently observed in different patients. Hence, individual tumour mutation profiles are dominated by the so-called *passenger* mutations which are highly variable between cancers and patients [39]. The development of next-generation sequencing (NGS) technologies has made it possible to screen entire (coding) genomes for the detection of potential neo-antigens in a clinically applicable time-frame. *In silico* tools aiming at identifying neo-antigens more likely to constitute good immunotherapy targets are also under constant development [40–44].

The requirement of a personalized approach to target neo-antigens can be a time-consuming and onerous procedure. While this limitation could be circumvented by the targeting of recurrent mutations at driver genes such as *BRAF* and *KRAS*, accumulated evidence suggests that such mutations are seldom immunogenic [45,46]. In fact, this might be expected, as it would be unlikely that immunogenic mutations would be so often favoured by clonal selection during tumour progression. Another aspect complicating the targeting of neo-antigens relates to intra-tumour heterogeneity. The identification of neo-antigens requires that the tumour is sampled and further processed for nucleic acid isolation and sequencing. Several reports have identified sampling issues as a major limitation for a comprehensive characterization of somatic alterations in tumours [47,48]. On the other hand, cancer therapies, including immunotherapies, will probably be the most successful when targeting clonal alterations present in any part of a tumour mass [13]. Another caveat that must be considered is that neo-antigens, particularly the ones derived from point mutations, have very similar sequences to their wild-type counterpart. If amino acid substitutions at anchor residues do not affect the binding affinity to HLA molecules or if substitutions at core residues do not significantly alter the molecular properties of a peptide, the likelihood that high avidity TCRs are present in an autologous T-cell repertoire may be low. This supports a fundamental role for frameshift mutations as these have the potential to generate highly immunogenic peptides [49]. However, frameshifts are notoriously difficult to detect, particularly in NGS data, and the capacity to identify them varies greatly between research groups.

## 3. The state of the art in cancer immunotherapy

T cells are key players in anti-tumour immunity and, therefore, the bulk of cancer immunotherapy research has focused on inducing T-cell-mediated anti-tumour responses. The current breakthrough in cancer immunotherapy results from the identification and subsequent targeting of checkpoint mechanisms in T cells with antibodies against CTLA-4, PD-1 and PD-L1 [50–53]. CTLA-4 and PD-1 are co-inhibitory receptors found on the cell surface of T cells. Upon binding to their corresponding ligands (CD80/86 and PD-L1/-L2, respectively), T cells become anergic: a physiological mechanism of peripheral tolerance or halting of inflammatory responses [54]. In the context of the tumour microenvironment, the aberrant expression of immune checkpoint ligands (on tumour and immune cells), together with chronic exposure to tumour antigens, can lead to the undesirable suppression of T-cell activity [55]. The blocking of such mechanisms can therefore unleash a renovated anti-tumour immune response. Moreover, checkpoint blockers were found to broaden the target of cytotoxic T-cell responses in cancer patients [56,57].

Treatment with checkpoint blocking antibodies has been approved for a number of cancers including melanoma, urothelial bladder cancer, head and neck squamous cell carcinoma, non-small cell lung cancer and classical Hodgkin lymphoma, while positive indications has been found for many other malignancies [50,58–62]. Immune checkpoint blockade has been shown to be most effective in tumours with high mutation burden that arises either from chronic exposure to DNA-damaging agents (e.g. smoking and ultraviolet radiation) or as a consequence of intrinsic DNA repair defects [16,17,63]. Accordingly, clinical responses have also been correlated with the mutation burden of tumours derived from the same organ [16,17,62]. Notably, pembrolizumab, an anti-PD-1 antibody, constitutes the FDA's first tissue/site-agnostic, molecular-guided approval as it is indicated for advanced mismatch repair-deficient cancers. These findings support the central role of neo-antigens in the therapeutic responses to immune checkpoint blockers. Nevertheless, the majority of patients with the so-called hypermutated tumours do not respond to checkpoint blockade and the ability to predict responses by discovering additional biomarkers is a major focus of research in the field [64]. In order for CD8^+^ T cells to fulfil their cytotoxic activity, they must infiltrate tumour tissues and subsequently recognize cancer antigens loaded on HLA class I molecules. Therefore, defects in the antigen processing and presentation machinery are often observed as immunoediting phenotypes in tumour cells [65–69]. Additionally, tumour cells can escape cytokine-mediated immune responses by mutating components of the IFN-γ pathway. Metastatic melanoma patients that did not respond to CTLA-4 treatment were found to have tumours with genetic defects in *IFNGR1/2*, *IRF1* and *JAK2* [70]. Similarly, melanoma and MMR-deficient colorectal cancer patients were found to be resistant to anti-PD-1 treatment due to inactivating mutations in *JAK1/2* [71,72]. Neo-antigen availability can also change in a tumour, due to clonal selection by immunoediting, enforced by neo-antigen-specific T cells [73,74].

Spontaneous, neo-antigen-driven, anti-tumour responses arise in many cancer patients, as demonstrated by the isolation of neo-antigen-reactive tumour-infiltrating lymphocytes (TIL) [75]. Furthermore, the presence of TIL, particularly with a type 1 inflammatory profile (i.e. IFNγ/IL-2-driven immune responses), is generally associated with an improved prognosis in cancer patients [76,77]. One approach to boost an autologous lymphocyte-mediated anti-tumour response is through adoptive T-cell transfer (ACT), which relies on the *ex vivo* expansion of tumour-reactive T cells and their reinfusion back in the patient [78]. The infusion product can consist of TIL or peripheral blood-derived tumour-specific T cells that are expanded in the presence of tumour cells or tumour antigens [79,80]. ACT-based treatments have produced some encouraging results, particularly for metastatic melanoma patients [73,80–83]. Verdegaal *et al*. reported on the successful treatment of a metastatic melanoma patient with CD4^+^ and CD8^+^ tumour-specific T cells [73,80]. In a fascinating example, the potency of neo-antigen-specific ACT is illustrated by the treatment of a metastatic cholangiocarcinoma patient, treated with a neo-antigen-reactive CD4^+^ T-cell product derived from TIL, resulting in stable disease [82]. These findings underscore the relevance that ACT might have for some patients, but similar to for checkpoint blockade, there is a need to discover biomarkers that indicate *a priori* which patients may benefit from it.

Today, many ongoing clinical trials are investigating the clinical effect of combining different immunotherapies. The use of anti-CTLA-4 in addition to anti-PD-1 antibodies resulted in increased overall survival rates in previously untreated melanoma patients [84,85]. Furthermore, other immune regulators, such as LAG-3, TIM-3, ICOS or NKG2D are promising new therapeutic targets [86–90]. Additional research will be important to address resistance to first-generation immune checkpoint blockers as, for instance, LAG-3 and TIM-3 upregulation is observed following anti-PD-1 treatment [86]. Likewise, CD137 co-stimulation is studied for its synergistic effects with ACT [91,92]. Finally, checkpoint blockade therapies may also be used in combination with standard chemo- and radiotherapy interventions which are known to enhance tumour immunogenicity [93,94].

Other avenues like therapeutic vaccination with synthetic peptides corresponding to neo-antigens are being explored. This strategy aims to prime autologous T cells from cancer patients against tumour-specific antigens to unleash anti-tumour immune responses. In addition to providing neo-antigens as immunotherapy products, several co-stimulatory factors are needed to induce an effective anti-tumour T-cell response [95], including provision of danger signals by adjuvants and/or homing of cellular-based vaccines [96–98]. Encouraging clinical responses were obtained with neo-antigen-based peptides plus polyICLC vaccinations in previously untreated metastatic melanoma patients [99]. This intervention was shown to induce CD4^+^ and CD8^+^ anti-tumour T-cell responses against several epitopes. Four out of six patients had no recurrence after 25 months; two patients with tumour recurrence received subsequent anti-PD1 therapy leading to complete tumour regression [99]. In another phase I study, stage III melanoma patients pre-treated with ipilimumab and by surgical resection received a vaccine consisting of autologous dendritic cells presenting neo-antigens that were determined by sequencing [100]. Both vaccination strategies induced tumour-directed immune responses with concomitant broadening of the targeted antigen repertoire without inducing side-effects [99,100]. Nevertheless, to date, the number of vaccination studies involving neo-antigens that reported positive clinical outcomes is limited. This might be explained by the fact that the bulk of this research, in previous decades, has focused on targeting oncogenes and tumour suppressors (e.g. *TP53*) with recurrent mutations [101]. Therefore, these studies did not consider the largest source of neo-antigens in tumour—*passenger* mutations.

The requirement that neo-antigens are presented in complex with HLA class I hinders the widespread application of neo-antigen-targeted therapies in the form of peptide vaccination or ACT. Therefore, CAR T cells were designed to enable the targeting of any cell surface molecule, in an HLA non-restricted fashion [9]. This strategy has been particularly successful for treating haematological malignancies, because highly tissue/cell-restricted antigens are present on their easily accessible cells of origin [10–12]. In 2010, the first successful CAR T-cell therapy was reported in a lymphoma patient who was pre-treated with chemotherapy [10]. The infusion product consisted of autologous T cells transduced with retroviruses encoding the variable region of the anti-CD19, B-cell antigen, which was joined to part of the co-stimulatory CD28 molecule and CD3*ζ* signalling domain for T-cell activation. Investigations in larger cohorts showed clinical responses [102,103], but severe side-effects arose, including treatment-related deaths [104–106]. These side-effects derive from high cytokine concentrations (cytokine storm), produced by the infused engineered T cells that become hyper-activated as a result of high affinity of their receptor to the target molecules. Recently, two second generation CAR therapies targeting CD19 have been approved by the FDA for treatment of patients with relapsed/refractory diffuse large B-cell lymphoma and relapsed/refractory B-cell precursor acute lymphoblastic leukaemia [107,108]. In search for optimal effectivity and specificity, third generation CARs are currently being developed, which contain two co-stimulatory domains [109–112]. Furthermore, investigations are ongoing to improve the treatment of haematological diseases while limiting the severity of side-effects, as well as investigations on the clinical efficacy of genetically engineered T cells in solid tumours [109,113,114]. The targeting of the latter has proved to be particularly challenging and complicating factors include the identification of specific, targetable antigens and the homing of CAR T cells to the tumour tissues where in turn they are exposed to a complex tumour microenvironment [115]. On the other hand, CAR T cells are a very attractive tool to treat cancers arising in non-vital organs where specific antigens are expressed (e.g. thyroid and ovaries).

## 4. The immune landscape of low mutation burden tumours

As discussed, neo-antigens constitute attractive targets for immunotherapy and clinical responses with checkpoint blockers have been correlated to the mutation burden of tumours [16,62]. Cancers with 10 mutations/Mb or more have been proposed as susceptible for checkpoint blockade, indicating the importance of neo-antigen presence for a potent immune response [116]. However, not all patients with high mutation burden tumours benefit from these therapies, and the precise determinants of response are undefined at the moment. Furthermore, the division between tumours with high, moderate and low mutation burden is somewhat arbitrary. In theory, tumours with low/moderate mutation burden that present neo-antigens in complex with HLA class I could still be eligible for T-cell-mediated immunotherapy. However, several questions remain unanswered: does the low number of neo-antigens translate to the improbability that a neo-antigen ‘survives’ the antigen processing machinery? On the other hand, if a small number of neo-antigens is indeed presented by a tumour cell, is it enough to provoke an inflammatory response that is required for tumour elimination?

Medulloblastoma, the most common brain tumour in children, has a low mutation burden, but was found to upregulate IDO1 expression [117]. IDO1 enhances immunosuppressive effects leading to an increase of Tregs and dampened activity of effector T cells [118]. Therefore, upregulation of IDO1 can be classified as an immune escape mechanism, indicating a role for the immune system in the control of medulloblastoma progression. Additionally, acute myeloid leukaemia (AML) cells are known to overexpress PD-L1 [119] and IDO1 [120], and AML blasts can secrete arginase II in order to promote immune escape by suppressing T-cell proliferation and polarizing monocyte differentiation towards an M2 phenotype [121]. Another tumour with low/moderate mutation burden, Hodgkin lymphoma, is characterized by few tumour cells and many immune cells that are attracted by the tumour-secreted cytokines [122]. However, these tumour-infiltrating immune cells display an immunosuppressive rather than anti-tumourigenic phenotype [122]. Immunotherapies are regularly employed to treat this disease, including antibodies targeting CD20, CD30 and checkpoint inhibitors targeting PD-1 [7,61,123]. Effectiveness of the latter may reside in the genetic overexpression of PD-L1 by the tumour cells [122]. TILs in Hodgkin lymphoma were found to express low levels of PD-1, but the blockade of this co-inhibitory mechanism was shown to result in an enhanced anti-tumour activity [61]. This finding underlines the existence of a T-cell-mediated anti-tumour response, which might be circumvented by the tumour through PD-L1 expression. Nevertheless, the immune evasive mechanisms observed in AML and Hodgkin lymphoma are probably closely connected to the function of their precursor cells and the persistent interaction of these pathologies with the immune system. A last example of a tumour type with low/moderate mutation burden that has potential for treament with immunotherapeutic strategies is renal cell carcinoma (RCC). Sensitivity to immunotherapeutic intervention in this tumour type was already known from the clinical responses of some RCC patients to IL-2 treatment [124]. Recently, patient overall survival was shown to increase from 19.6 to 25 months with anti-PD-1 therapy compared to standard care with the mTOR inhibitor everolimus [125]. The underlying mechanisms making this tumour susceptible for immunotherapeutics are not understood yet, but the composition of the tumour microenvironment might play an important role. High lymphocyte infiltration was found to correlate with high risk for disease progression, which is a paradox characteristic of RCC. This might relate to the exhausted phenotype of infiltrating lymphocytes which contributes to an immunosuppressive microenvironment [126]. Furthermore, neo-antigen depletion due to immune selection was demonstrated to occur in RCC and a positive correlation was observed between mutations in the antigen-presenting machinery and cytotoxic activity by immune cells, suggesting the presence of ongoing anti-tumour immune reactions [67]. Finally, RCC was found to have the highest number of frameshift mutations out of 19 different cancer types, which might explain the immunogenicity observed in these tumours despite their moderate total mutation burden [49]. These examples of tumours with low mutation burden presenting susceptibility to immunotherapeutic strategies indicate the existence of autologous tumour-specific T cells with the potential to recognize (neo-) antigens, even when present in small numbers.

## 5. Immunotherapies for tumours with low mutation burden

Previous works by Tran *et al*. [82,127] support the idea that most tumours present neo-antigens and that these can be targeted by the immune system, e.g. gastrointestinal cancers with low and moderate mutation burden including a cholangiocarcinoma patient with only 26 non-synonymous mutations. Therefore, the clinical applicability of neo-antigen-targeted ACT or peptide-based vaccination strategies for low mutation burden tumours should be explored. The detection rate of autologous T-cell reactivity to neo-antigens is often described to be approximately 1% of the non-synonymous mutations that are transcribed in a tumour [83,127,128]. Currently, NGS is regularly used to determine neo-antigen presence, but improvements in capture methods for targeted panels (e.g. exome) and mutation detection algorithms might enhance the initial pool of targetable mutations in tumours with low mutation burden. For these, the use of *in silico* prediction models for antigen processing and HLA binding affinity might not be necessary for a first T-cell reactivity screening using long peptides, because the number of mutations is low and all neo-antigens can be tested for their ability to induce T-cell activation. However, to directly investigate T-cell reactivity against short peptides, *in* silico tools are still required.

**Figure 1.**
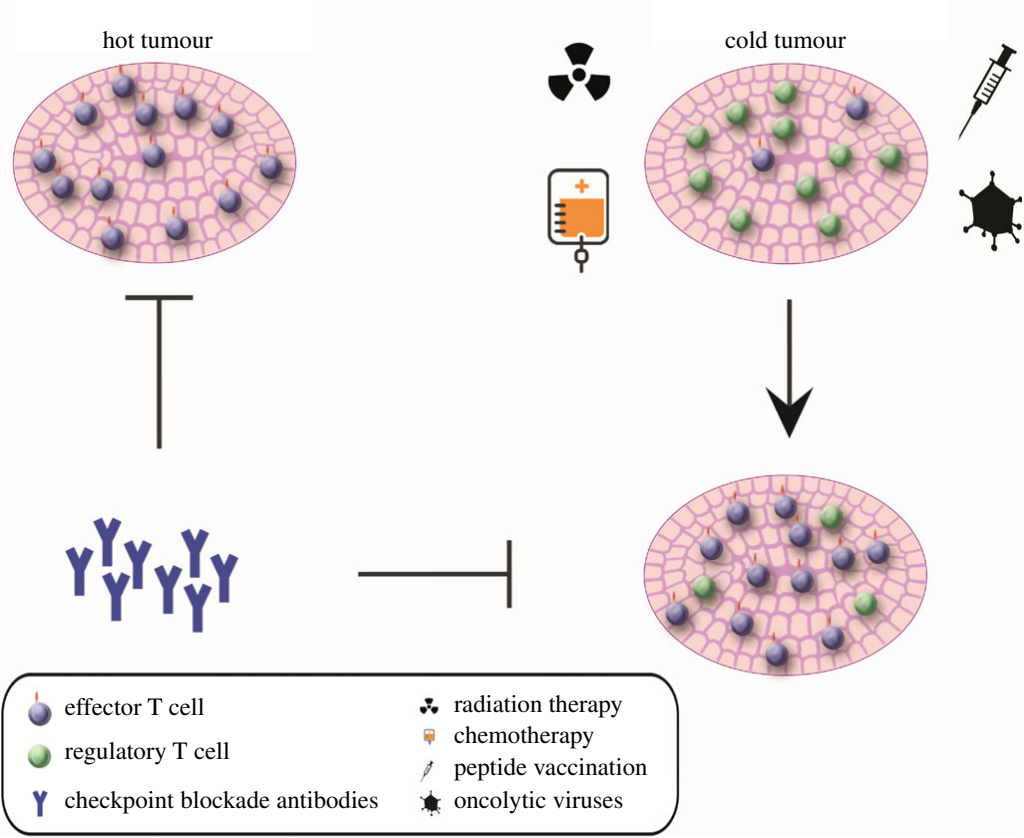
(Immuno) therapeutic strategies in tumours with ‘hot’ and ‘cold’ immune microenvironments. Checkpoint blockade therapies are mostly applicable to ‘hot’ tumours which present an inflammatory profile as a consequence of their high mutation burden. We propose that ‘cold’ tumours might be sensitized to checkpoint blockade if this is used in combination with radiotherapy, chemotherapy, peptide vaccination or oncolytic viruses, to boost anti-tumour immune responses.

Immunotherapies have a high synergistic potential with standard chemo- and radiotherapies as these are known to induce immunogenic cell death [129,130]. This synergy might be especially valuable for tumours with low mutation burden which do not respond to immunotherapy alone, and which could benefit from the transformation of a ‘cold’ immune microenvironment into a ‘hot’ microenvironment with an inflammatory profile (figure 1) [131,132]. The rationale of classical chemotherapy and radiotherapy encompasses the targeting of fast-dividing tissues by impairing mitosis and inducing DNA damage. This leads to the release of tumour antigens and damage-associated molecular patterns which activate APCs [133]. Macrophages are attracted to consume the damaged tumour cells, which further enhances the anti-tumour response of T cells upon presentation of the tumour antigens [134]. In addition, radiotherapy leads to the release of nuclear DNA in the cytoplasm, activating the stimulator of interferon genes (STING) pathway, which is a direct link between the innate immune system and DNA damage [135,136]. Furthermore, the tumour microenvironment is disrupted by chemoradiation, thereby disturbing the immune suppressive milieu in tumours. This includes increased antigen presentation and expression of co-stimulatory molecules as well as inhibition of regulatory T-cell and myeloid-derived suppressor cell function [129,137–139]. In melanoma patients, an improved clinical response rate was observed upon treatment with a combination of anti-CTLA-4/PD-1 with radiotherapy, compared to treatment without radiation [130]. Moreover, combined radiotherapy with anti-CTLA-4 treatment induced abscopal effects (shrinkage of tumour lesions outside of the target region of radiotherapy), in this case consisting of complete regression of metastases at different sites [140]. Similarly, abscopal effects were observed in a treatment-refractory metastatic lung adenocarcinoma patient after therapy with radiotherapy and ipilimumab [141]. Tumours treated pre-surgically with neo-adjuvant therapy might be particularly interesting for the investigation of the synergistic effect of chemoradiation and immunotherapy in cancers with low mutation burden. Among these, rectal cancers and oesophageal tumours are excellent candidates for clinical trials aiming at reducing mortality and treatment-related morbidity.

Another avenue that may lead to the sensitization of additional tumours to immunotherapeutic intervention is epigenetic modulation of cancer cells [142]. Epigenetic regulation is fundamental for gene expression and, consequently, for neo-antigen availability. Furthermore, in order to evade the immune system, tumours might acquire epigenetic footprints that change the expression of immunomodulatory genes. For instance, the expression of specific HLA alleles, with affinity to neo-antigens, can be suppressed in tumour cells due to epigenetic changes [143,144]. Such observations are strongly supportive of adopting epigenetic modifiers to restore or improve immunogenicity of some cancers [145]. More specifically, epigenetic modifiers have been shown to increase CD8^+^ T-cell infiltration in ovarian cancer and the immunogenicity of colorectal cancer cells was increased upon treatment with DNA-demethylating agents [146,147]. Epigenetic drugs could thus tackle the heterogenic expression of, among others, HLA molecules and neo-antigens, thereby enhancing anti-tumour immunity.

Another obstacle to employing immunotherapies for the treatment of tumours with low mutation burden relates to the fact that they are usually poorly infiltrated by immune cells. The initiation of an adaptive anti-tumour immune response probably relies on a robust inflammatory trigger that is absent in poorly immunogenic tumours. On the other hand, such inflammatory threshold in tumours with high mutation burden is most likely reached due to the abundance of mutated antigens. A strategy to artificially induce an inflammatory response that complements immunotherapeutic approaches is oncolytic virotherapy (figure 1). Talimogene laherparepvec, a genetically engineered herpes virus, replicates specifically in cancer cells and induces tumour cell death [148]. It was also shown to induce the expression of GM-CSF in tumours, which attracts dendritic cells that take up tumour antigens after cancer cell death. A phase Ib clinical trial obtained objective response rates (62%) and complete response rates (33%) in advanced melanoma patients, which were treated with a talimogene laherparepvec vaccination combined with pembroluzimab (anti-PD-1 blocker) [149]. The vaccination treatment was shown to induce infiltration of T cells that often expressed PD-1, especially in otherwise non-infiltrated ‘cold’ tumours, explaining the patients' sensitivity to PD-1 blockade. While such combination therapies were mainly performed in immunogenic tumours, their success and rationale supports the investigation of their applicability in tumours with low mutation burden.

## 6. Concluding remarks

Immunotherapy, particularly checkpoint blockade, can induce robust and durable anti-tumour responses in a significant proportion of patients, predominantly when applied for the treatment of cancers with high mutation burden. Until today, the applicability of these treatments for other cancer types is very limited. During the last decade, different groups have demonstrated the possibility of identifying neo-antigen-targeted immune cell repsonses in tumours with intermediate/low mutation burden. Recent work in our laboratory confirms that neo-antigen-reactive T cells are present in low mutation burden, mismatch repair-proficient colorectal carcinomas (van den Bulk *et al.* 2018, unpublished data). These findings underscore the relevance of developing neo-antigen targeting immunotherapies for low mutation burden tumours by tuning anti-tumour inflammatory responses. ‘Cold’, poorly immunogenic, tumours will require rationale-based interventions that make use of combinatorial therapies, including radio/chemotherapy or oncolytic viruses, to switch cancer immune microenvironments to a ‘hot’ state.

## Data accessibility

This article has no additional data.
